# Supplementary material for: The cryo-EM structure of the SNX–BAR Mvp1 tetramer
Source: Nat Commun. 2020 Mar 20;11:1506. doi: 10.1038/s41467-020-15110-5 (PMC7083883; doi:10.1038/s41467-020-15110-5)
Supplement: Supplementary file 1 — Supplementary Information [file 41467_2020_15110_MOESM1_ESM.pdf]

Supplementary Information

for

The cryo-EM structure of the SNX-BAR Mvp1 tetramer

Dapeng Sun, Natalia V. Varlakhanova, Bryan A. Tornabene, Rajesh Ramachandran, Peijun Zhang[‡] & Marijn G. J. Ford[‡]

This document includes

Supplementary Tables 1-7

Supplementary Figures and Legends 1-6

Supplementary References

Supplementary Tables

Supplementary Table 1. Genetic interaction profile similarity network for *MVP1*, cut-off at a Pearson's Correlation Coefficient (PCC) of 0.2. The data were extracted from thecellmap.org. Functions for the listed ORFs were manually assigned.

Supplementary Table 2. Yeast strains used in this work.

Supplementary Table 3. Plasmids used in this work.

Supplementary Table 4. Primers used in this work.

Supplementary Table 5. Cryo-EM data collection statistics

Supplementary Table 6. Cryo-EM data processing statistics

Supplementary Table 7. Model statistics

<u>Supplementary Table 1:</u> Genetic interaction profile similarity network for Δ*mvp1*

ORF	Allele	Correlation PCC	Annotations
YHR012W	*vps29Δ*	0.365	RETROMER
YJL154C	*vps35Δ*	0.318	RETROMER
YOR070C	*gyp1Δ*	0.311	GOLGI Trafficking
YJL053W	*pep8Δ*	0.301	RETROMER
YDL145C	*cop1-1*	0.295	Coatomer
YBR164C	*arl1Δ*	0.281	GOLGI Trafficking
YLR360W	*vps38Δ*	0.267	Production of PI3P
YPL051W	*arl3Δ*	0.257	GOLGI Trafficking
YOR068C	*vam10Δ*	0.252	Vacuolar fusion
YJL004C	*sys1Δ*	0.248	GOLGI Trafficking
YGR120C	*cog2-1*	0.247	GOLGI Trafficking
YDR320C	*swa2Δ*	0.241	Vesicular transport
YOL018C	*tlg2Δ*	0.236	GOLGI Trafficking
YPL120W	*vps30Δ*	0.235	Production of PI3P
YNL051W	*cog5Δ*	0.231	GOLGI Trafficking
YFR051C	*ret2-1*	0.225	Coatomer
YOR069W	*vps5Δ*	0.223	RETROMER
YGL005C	*cog7Δ*	0.223	GOLGI Trafficking
YFL038C	*ypt1-3*	0.222	GOLGI Trafficking
YDR456W	*nhx1Δ*	0.222	Vacuolar osmoregulation
YNL041C	*cog6Δ*	0.220	GOLGI Trafficking
YPR105C	*cog4-5004*	0.213	GOLGI Trafficking
YOR132W	*vps17Δ*	0.210	RETROMER
YDR108W	*trs85Δ*	0.209	GOLGI Trafficking
YPL195W	*apl5Δ*	0.209	AP-3 adaptor
YDR137W	*rgp1Δ*	0.207	GOLGI Trafficking
YPL010W	*ret3-1*	0.206	Coatomer
YDR484W	*vps52Δ*	0.206	GARP endosome to Golgi
YMR274C	*rce1Δ*	0.204	Maturation of Ras
YML071C	*cog8Δ*	0.201	GOLGI Trafficking
YDR246W	*trs23-5001*	0.200	GOLGI Trafficking

<u>Supplementary Table 2:</u> Yeast strains used in this work

**Strain**	**Genotype**	**Reference**
W303A	*MAT***a**; *ade2-1*; *leu2-3,112*; *his3-11,15*; *trp1-1*; *ura3-1*; *can1-100*	
PY_225	*MAT***a**; *ade2-1*; *leu2-3,112*; *his3-11,15*; *trp1-1*; *ura3-1*; *can1-100*; Δ*mvp1::HIS3*	This work
PY_112	*MAT***a**; *ade2-1*; *leu2-3,112*; *his3-11,15*; *trp1-1*; *ura3-1*; *can1-100*; Δ*vps34::HIS3*	This work

<u>Supplementary Table 3:</u> Plasmids used in this work

**Plasmid**	**Details**	**Reference**
MVP1-EGFP	pRS315 *S cer. MVP1*-yEGFP	This work
VPS10-yEGFP	pRS315 *S cer*. *VPS10*-yEGFP	This work
MVP1	pRS314 *S cer*. *MVP1*	This work
MVP1 Mut1-EGFP	pRS314 *S cer. MVP1* K198-I200->AAA-yEGFP	This work
MVP1 K198A-EGFP	pRS314 *S cer. MVP1* K198A-yEGFP	This work
MVP1 R198A, I200A-yEGFP	pRS314 *S cer. MVP1* R199A, I200A-yEGFP	This work
MVP1 Mut1	pRS314 *S cer. MVP1* K198-I200->AAA	This work
MVP1 R198A, I200A	pRS314 *S cer. MVP1* R199A, I200A	This work
MVP1 K198A	pRS314 *S cer. MVP1* K198A	This work
MVP1 Δ2-100-yEGFP	pRS314 *S cer. MVP1* Δ2-100-yEGFP	This work
MVP1 Δ2-100	pRS314 *S cer. MVP1* Δ2-100	This work
Mvp1	pET-15b *S cer*. *MVP1*	This work
Mvp1-PreScission-MBP	pMW-PreScission-MBP *S cer. MVP1*	This work
Mvp1 Δ2-78	pET-15b *S cer. MVP1* Δ2-78	This work
Mvp1 Δ2-100	pMW-PreScission-MBP *S cer. MVP1* Δ2-100	This work
Mvp1 PreScission at 99	pET-15b *S cer*. *MVP1* 1-99-PreScission-100-end	This work
Mvp1 K198A	pET-15b *S cer*. *MVP1* K198A	This work
Mvp1 R199A, I200A	pET-15b *S cer*. *MVP1* R199A, I200A	This work
Mvp1 Mut1	pET-15b *S cer*. *MVP1* K198-I200->AAA	This work

MVP1-yEGFP, mutant variants and *VPS10*-yEGFP are all followed by the terminator from *VPS1*, which consists of the 300 nucleotides (nt) immediately downstream of the *VPS1* ORF stop codon. The *MVP1* promoter and terminator are 250 and 150 nt, respectively. The *VPS10* promoter is 450 nt.

Supplementary Table 4: Primers used in this work

Primer	Sequence (5'-3')
Δ*mvp1::HIS3* F (MF_1025)	TTGGATTCTATAAAACACCACTGAGGCGAAAAAAAAGTACGGATCCCCGGGTTAATTAA
Δ*mvp1::HIS3* R (MF_1026)	GTTTTGGCAGACTAAGTGGTTAGTCTTCACTCCGTAAAATGAATTCGAGCTCGTTTAAAC
Δ*vps34::HIS3* F (MF_350)	CATCTCCGTGAAGCATTGAGGGAAGGGTTTAACTCCAACACGGATCCCCGGGTTAATTAA
Δ*vps34::HIS3* R (MF_350)	GTGACGAAATTTAAATTTTGAAGCACCAATTATCAACCAAGAATTCGAGCTCGTTTAAAC
MVP1 promoter (250 nt) F (MF_1027)	CCCCCCCTCGAGGTCGACGGTATCGATATTAAGACACTTTTACTTTTCTCTACGC
MVP1 terminator (150 nt) R (MF_1095)	CGACTCACTATAGGGCGAATTGGAGCTCTTGAAAAACAAATAAATAACAATTTATAGTG
VPS1 terminator (300 nt) R (MF_30)	CGACTCACTATAGGGCGAATTGGAGCTCATAGTACCTTATTTAGTATTGGACCATTG
Linker into EGFP F (MF_506)	CGACGGATCCCCGGGTTAATTAACTCTAAAGGTGAAGAATTATTCACTGG
MVP1 CDS into Linker R (MF_1028)	CCCGGGGATCCGTCGACCTTCACGAGAAATCGGC
VPS10 promoter (540 nt) F (MF_1038)	CCCCCCCTCGAGGTCGACGGTATCGATTGAATGAACACCAAAAGATGTGTG
VPS10 CDS into Linker F (MF_1036)	ATCGACAGGCCTGATTCTACAGCGCCATCTAACGAAAACCAGGGTGACGGTGCTGGTTTA
VPS10 CDS into Linker R (MF_1039)	GTTAATTAAACCAGCACCGTCACCCTGGTTTTCGTTAGATGGCGCTG
MVP1 K198A F (MF_1159)	CCTGAGCTACCTCCCgcaAGAATTGGATCCCAAAACGC
MVP1 K198A R (MF_1159)	GCGTTTTGGGATCCAATTCTtgcGGGAGGTAGCTCAGG

MVP1 R199A, I200A F (MF_1151)	CCTGAGCTACCTCCCAAAgcagctGGATCCCAAAACGC
MVP1 R199A, I200A R (MF_1151)	GCGTTTTGGGATCCagctgcTTTGGGAGGTAGCTCAGG
MVP1 Mut1 K198-I200->AAA F (MF_1073)	CCTGAGCTACCTCCCgcagcagctGGATCCCAAAACGC
MVP1 Mut1 K198-I200->AAA R (MF_1074)	GCGTTTTGGGATCCagctgctgcGGGAGGTAGCTCAGG
MVP1 Δ2-100 F (MF_1146)	CACCACTGAGGCGAAAAAAAAAGTAATGGCTAGAGATCAGAATATAGAAGAATCTG
MVP1 Δ2-100 R (MF_1145)	CAGATTCTTCTATATTCTGATCTCTAGCCATTACTTTTTTTTTCGCCTCAGTGGTG
MVP1 CDS F (MF_917)	GCGGCCTGGTGCCGCGCGGCAGCCATATGGACAATTACGAAGGCAGTGATCC
MVP1 CDS R (MF_918)	CGGGCTTTGTTAGCAGCCGGATCCTTATTCACGAGAAATCGGCATATCC
MVP1 CDS F (MF_921)	CTTTAAGAAGGAGATATACATATGGACAATTACGAAGGCAGTGATCC
MVP1 into PreScission cleavage site R (MF_922)	CCTGGAACAGAACTTCCAGGCCGGATCCTTCACGAGAAATCGGCATATCC
PreScission cleavage site into MBP CDS F (mgjf_505)	CGCGGATCCGGCCTGGAAGTTCTGTTCCAGGGGCCCGGCATGAAAATCGAAGAAGGTAAACTGG
MBP R (mgjf_506)	CCGGAATTCGCTAGCCTACGAGCTCGAATTAGTCTGCGC
MVP1 Δ2-78 (MF_972)	GCCTGGTGCCGCGCGGCAGCCATATGGGTGCAACTGGCATGAGCCAAACTCC
MVP1 Δ2-100 F (MF_975)	CTTTAAGAAGGAGATATACATATGGCTAGAGATCAGAATATAGAAGAATCTG
MVP1 PreScission at 99 F (MF_1192)	GAGACAGTTATAGATAAGAATCTGGAAGTTCTGTTCCAGGGGCCCGATGCTAGAGATCAG
MVP1 PreScission at 99 R (MF_1193)	CTGATCTCTAGCATCGGGCCCCTGGAACAGAACTTCCAGATTCTTATCTATAACTGTCTC

As all cloning was performed by Gibson assembly or SOEing, primers frequently incorporate sequence from the destination vector. Where target vector sequence is incorporated, it is shown in blue. For primers used to generate cassettes for homologous recombination-mediated removal of yeast ORFs, the portion corresponding to sequence from pFA6a-His3MX6 is shown in blue. Sequence from EGFP is shown in green and sites of mutations are shown in lower case.

<u>Supplementary Table 5:</u> Cryo-EM data collection

**Data collection**	Mvp1	Mvp1 Mut1
Microscope	FEI Titan Krios	FEI Titan Krios
Magnification	75,000	75,000
Voltage (kV)	300	300
Defocus range (μm)	0.5-3.5	1.5-5.0
Pixel size (Å)	1.056	1.056
Electron dose ($e^-/Å^2$)	58	58
Micrographs (number)	1,620	1,350

Supplementary Table 6: Cryo-EM data processing

Data processing

Symmetry Imposed	C1	C2 top	C2 side	C2 end	D2
Particles (number)	82,000	97,000	91,000	115,000	49,000
Resolution (Å)	5.3	4.6	4.4	4.7	4.2
B-factor ($Å^2$)	-190	-210.7	-220	-234	-152.8
Accuracy of rotation (°)	2.98	2.83	2.88	3.37	2.933
Accuracy of translation (Å)	1.264	1.228	1.209	1.294	1.063

Supplementary Table 7: Model statistics

All-atom Contacts	Clashscore, all atoms	28.28	
	Poor rotamers	12	0.86 %
	Favored rotamers	1,222	87.29 %
	Ramachandran outliers	0	0.00 %
	Ramachandran favored	1,442	97.17 %
	MolProbity score	2.09	
	Cβ deviations >0.25 Å	0	
	Bad bonds	0 / 12724	0.00 %
	Bad angles	16 / 17,152	0.00 %
Peptide Omegas	Cis Prolines	0 / 48	0.00 %

Left column: Raw count. Right column: %

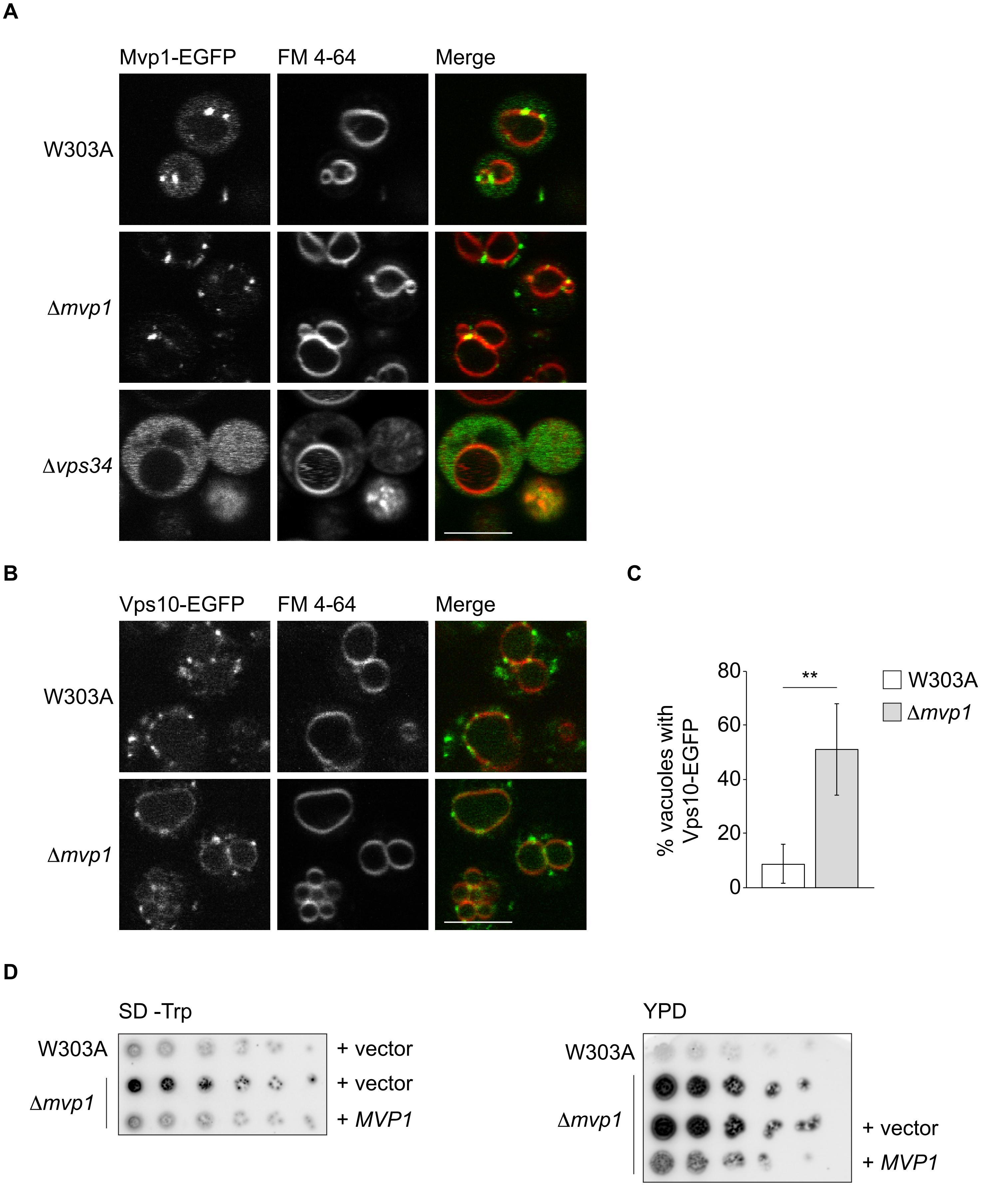

Supplementary Figure 1

Supplementary Fig. 1. **A.** Subcellular localization of EGFP-tagged Mvp1 when expressed in W303A, Δ*mvp1* and Δ*vps34* cells. Vps34 is the sole PI3 kinase in *S. cerevisiae.* Hence, Δ*vps34* cells lack PI3P. Vacuoles were stained with the lipophilic dye FM 4-64. Images were obtained by confocal microscopy. Representative cells are shown. Scale bar – 5 μm. **B.** Subcellular localization of Vps10 tagged with EGFP in W303A or Δ*mvp1* cells. Vacuoles were stained as in A. Scale bar – 5 μm. Note that in the Δ*mvp1* cells Vps10-EGFP localizes to the vacuolar membrane in addition to the puncta also seen in W303A cells. This additional vacuolar membrane localization represents a trafficking defect. **C.** Quantification of the results presented in B. Differences in means of % vacuoles in which membranes were labeled with Vps10-EGFP per field of cells were significant (two-sample t-test: 6 degrees of freedom, t=4.59 hence P=0.004). 113 and 91 vacuoles were assessed in W303A and Δ*mvp1* cells in total, respectively. **D.** CPY secretion assays. W303A or Δ*mvp1* cells expressing vector or *MVP1* were plated onto SD -TRP (left) or YPD (right) plates and incubated at 30°C for 24 h. Plates were then overlaid with nitrocellulose for an additional 16 h. CPY secretion was detected by immunoblotting using an anti-CPY antibody. The leftmost spot in each case is 2 μl of a OD_{600}=0.5 culture: spots to the right of this are sequential 5-fold dilutions. For C and D, source data are provided as a Source Data file.

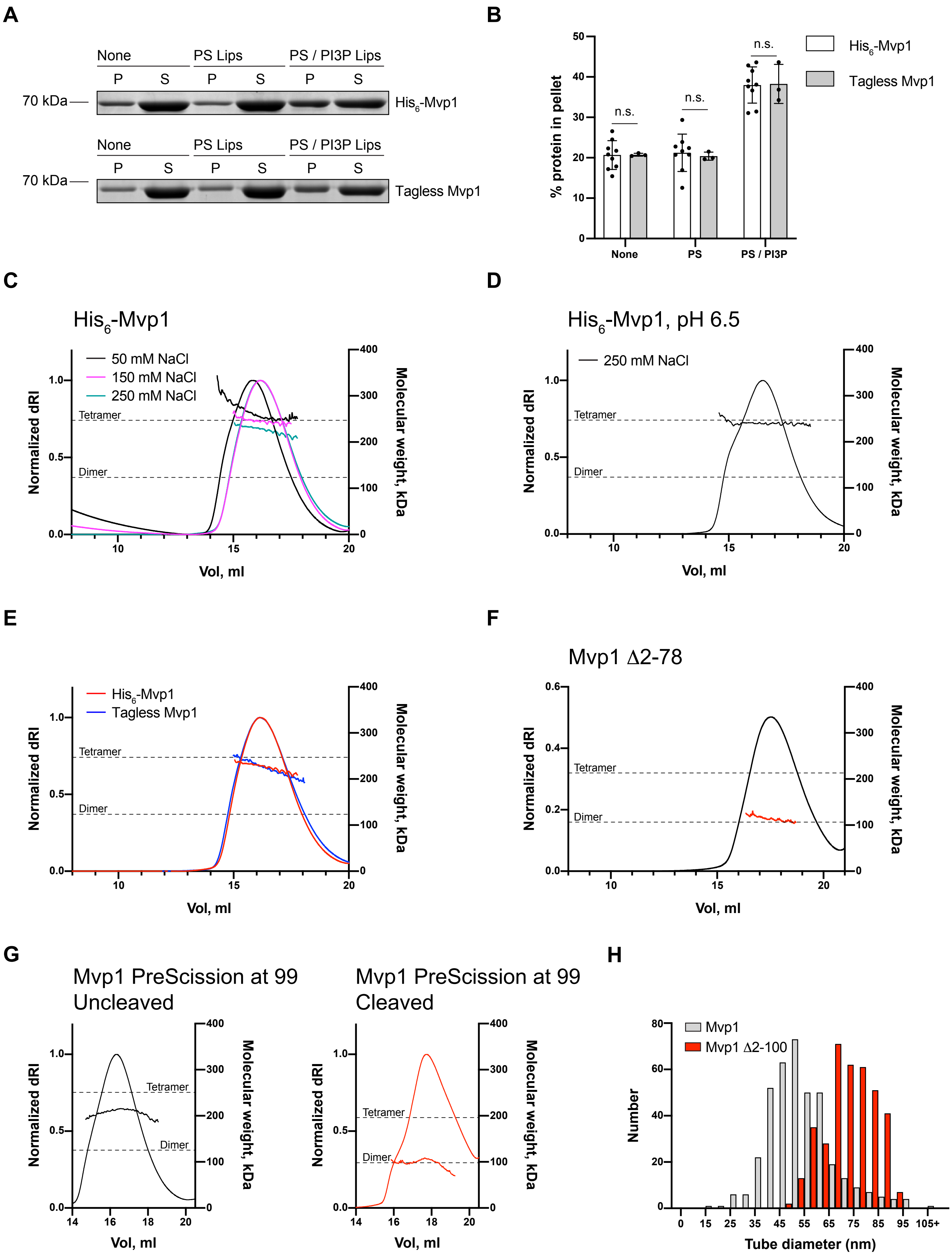

Supplementary Figure 2

Supplementary Fig. 2. A. Liposome binding of Mvp1 generated by either of two purification methods is the same. Comparison of liposome binding by His_6-Mvp1 and tagless Mvp1 (Mvp1 cleaved from Mvp1-PreScission-MBP). Protein (1.2 μM) was incubated without or with DOPS (PS) liposomes or PS liposomes supplemented with + 5% PI3P for 30 min at 21°C prior to sedimentation. Shown is a representative result. P – pellet; S – supernatant. Positions of molecular weight markers are shown to the left of the gels. **B.** Quantification of the results presented in A. Individual data points and mean ± s.d. are shown. n=9 for His6-Mvp1 and 3 for tagless Mvp1. A 3x2 factorial ANOVA was conducted to determine the effects of purification strategy (His_6-Mvp1 vs tagless Mvp1) or absence or presence of liposomes on the % sedimentation of protein. There was no significant interaction term (P=0.75) and only the liposome effect was significant (P<0.0001). There were no significant differences in liposome interaction with protein purified using either strategy. Please note that the data used for the quantification of tagless Mvp1 is the same dataset as used in Fig. 1E. **C.** Salt concentration effects on the absolute molecular weight of Mvp1, as determined using SEC-MALS. The respective molecular weights are plotted on the right-hand axis. Theoretical molecular weights of Mvp1 dimers and tetramers, calculated according to the Mvp1 sequence, are shown as dotted grey lines. Differential refractive indices of the elutions are plotted on the left-hand axis. Mvp1 was eluted in buffer containing 50 mM (black), 150 mM (magenta) or 250 mM (teal) NaCl. **D.** In a buffer of 20 mM MES pH 6.5, 250 mM NaCl, Mvp1 is fully tetrameric. SEC-MALS was performed as in C. **E.** Lack of effects of Mvp1 purification method on the absolute molecular weight of Mvp1, as determined using SEC-MALS. Data are presented as in C. His_6-Mvp1 (red) or tagless Mvp1 (blue) were eluted in buffer containing 250 mM NaCl. **F.** Mvp1 Δ2-78 is dimeric. Data are presented as in C. Mvp1 Δ2-78 was eluted in buffer containing 250 mM NaCl. **G.** Removal of the Mvp1 N-terminus results in Mvp1 dimers. A PreScission Protease site was engineered after residue 99 of the Mvp1 sequence. Uncleaved protein behaved similar to wild type at 250 mM NaCl (left panel). Cleavage with PreScission Protease generates dimers (right panel). The SEC-MALS was performed as in C. **H.** Histogram of diameters of tubes generated by Mvp1 (n=386) and Mvp1 Δ2-100 (n=371). Measurements were binned into classes of 5 nm prior to plotting. The

difference between medians (Mvp1: 48.0 nm; Mvp1 Δ2-100: 73.5 nm) was significant (Mann-Whitney: U = 12,536; P<0001). For A, B and H, source data are provided as a Source Data file.

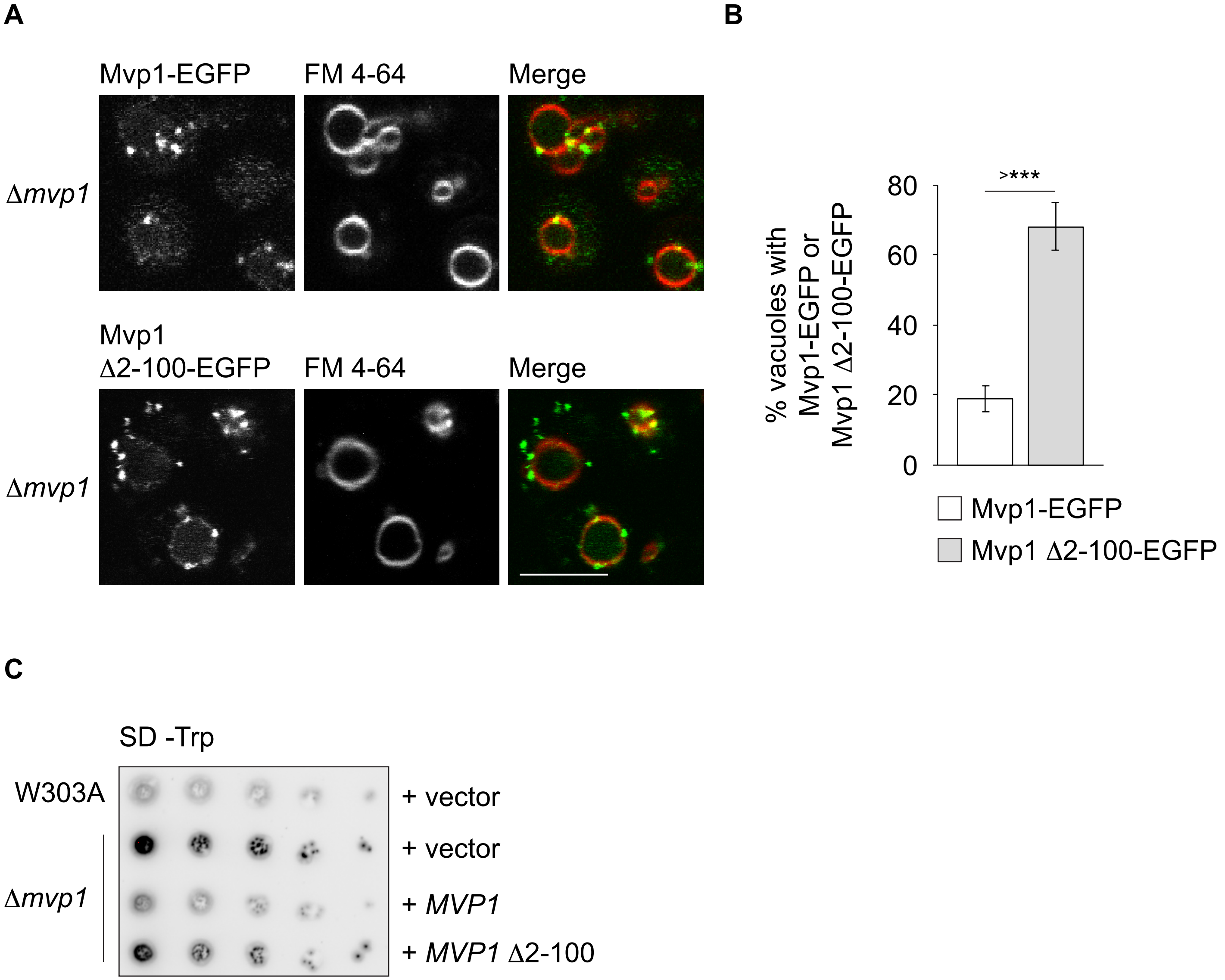

Supplementary Figure 3

Supplementary Fig. 3. A. Subcellular localization of EGFP-tagged Mvp1 or Mvp1 Δ2-100 when expressed in W303A or Δ*mvp1* cells. Vacuoles were stained with the lipophilic dye FM 4-64. Images were obtained by confocal microscopy. Representative cells are shown. Scale bar – 5 μm. **B.** Quantification of the results presented in A. Differences in means of % vacuolar membranes labeled with Mvp1-EGFP (*n* total = 206 vacuoles) or Mvp1 Δ2-100-EGFP (*n* total = 452 vacuoles), as appropriate, per field of cells were significant (two-sample t-test: 6 degrees of freedom, t=11.25 hence P=3E-5). **C.** The Mvp1 N-terminus is required for CPY sorting *in vivo*. W303A or Δ*mvp1* cells expressing vector, *MVP1* or *MVP1* Δ2-100 were plated onto SD -TRP plates and incubated at 30°C for 24 h. Plates were then overlaid with nitrocellulose for an additional 16 h. CPY secretion was detected by immunoblotting using an anti-CPY antibody. The leftmost spot in each case is 2 μl of a OD_{600}=0.5 culture: Spots to the right of this are sequential 5-fold dilutions. For B and C, source data are provided as a Source Data file.

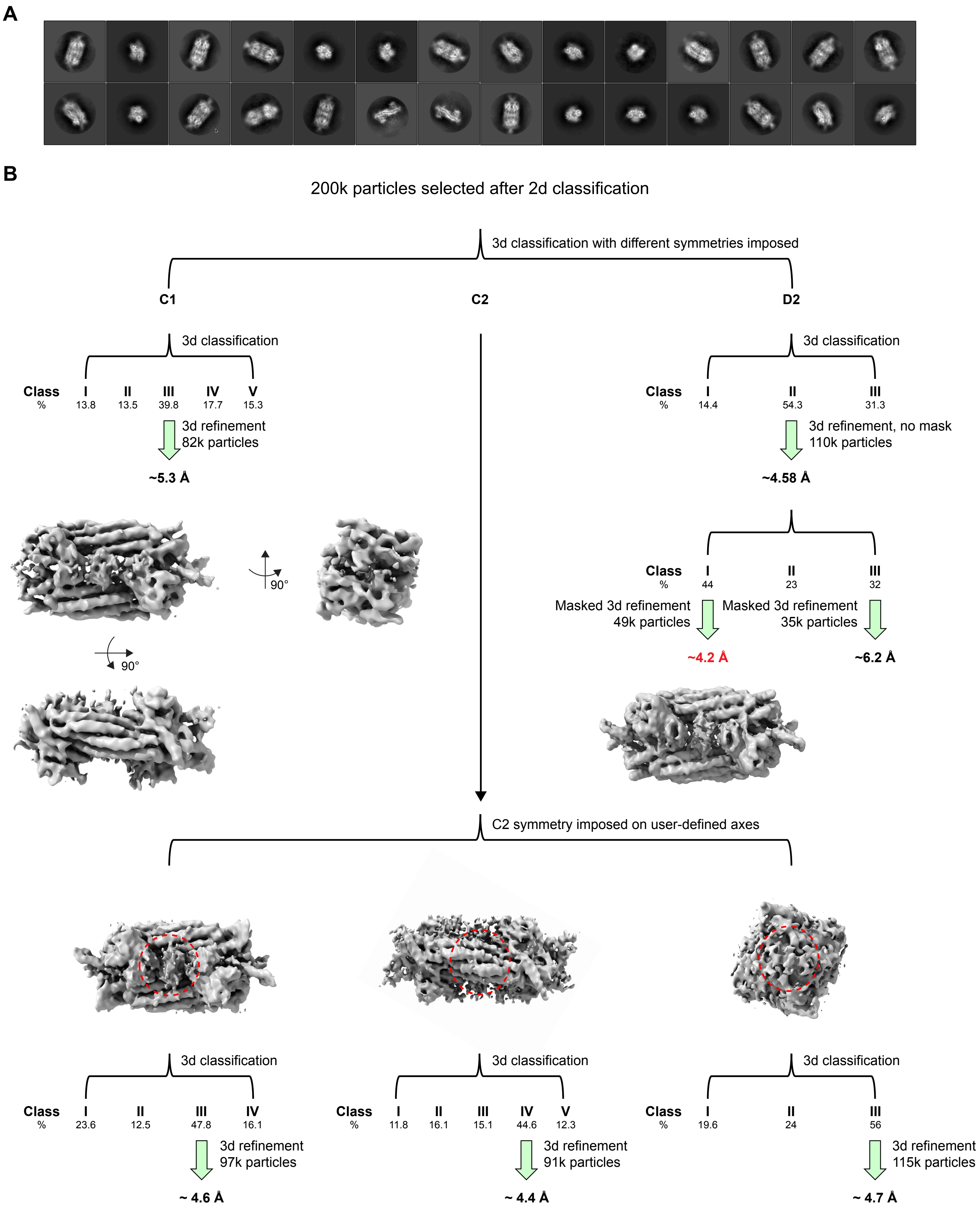

Supplementary Figure 4

Supplementary Fig. 4. 3d Mvp1 reconstruction pipeline. **A.** Representative 2d class averages of Mvp1 particles in different orientations. Note that some dimer classes are also present (see row 2, classes 6 and 7). **B.** Reconstruction workflow, detailing the strategies and particle cohort sizes used to generate the maps discussed in this work. Red dotted circles highlight the axis around which C2 symmetry was imposed.

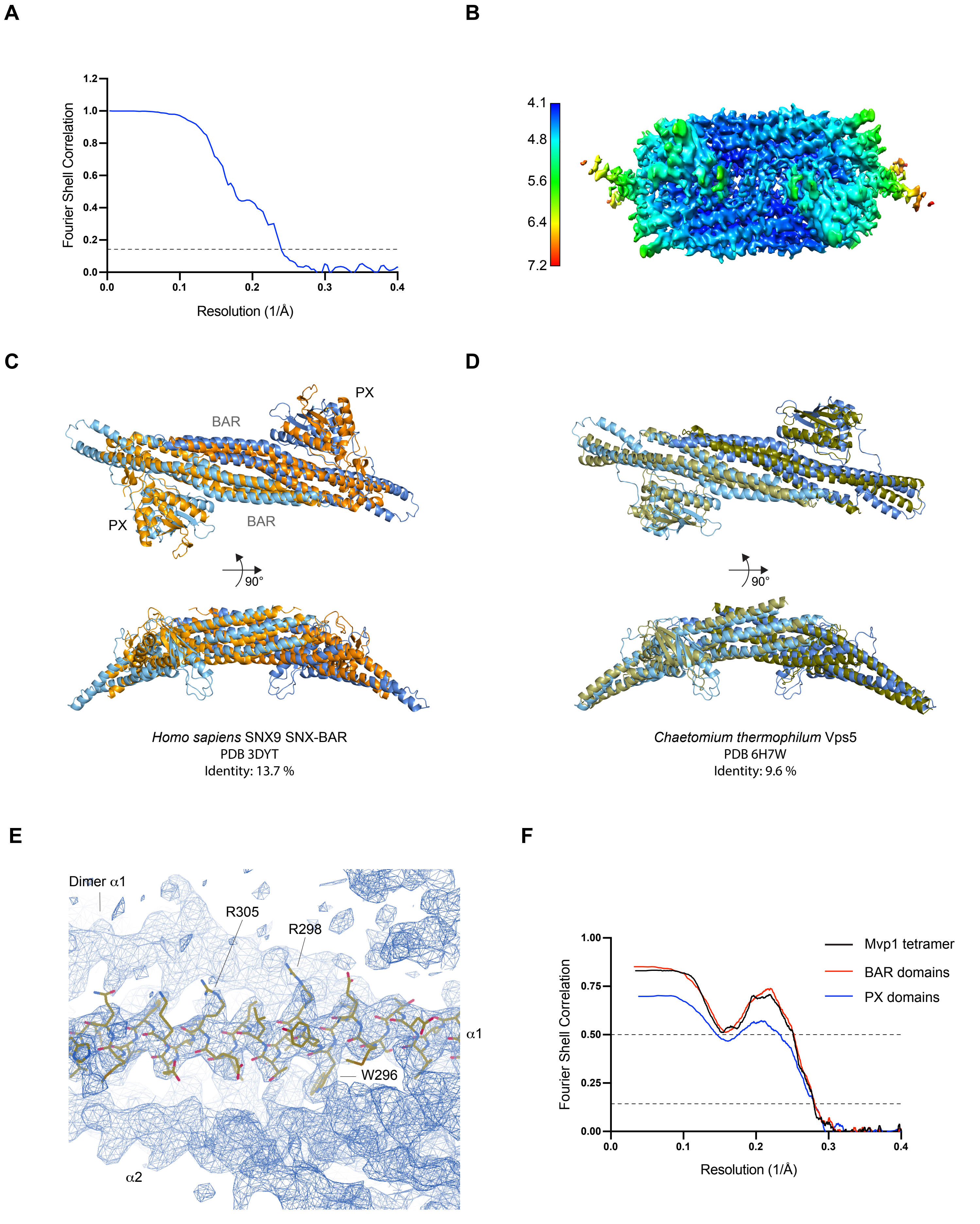

Supplementary Figure 5

Supplementary Fig. 5. Mvp1 structure determination details. **A.** Gold-standard Fourier Shell Correlation for the D2-symmetrized masked Mvp1 tetramer map. The curve intersects y=0.143 at a resolution of 4.2 Å. **B.** Local resolution of the D2-symmetrized map of the Mvp1 tetramer. **C** and **D.** SNX-BAR models used as a starting point for generating and completing the model of the Mvp1 tetramer. **C.** The *Homo sapiens* SNX9 SNX-BAR dimer (PDB 3DYT), with each chain colored in a different orange hue and superposed on an Mvp1 dimer from the Mvp1 tetramer (blues). **D.** The *Chaetomium thermophilum* Vps5 (PDB 6H7W) dimer, colored in greens, again superposed onto the Mvp1 dimer. **E.** Example of the D2-symmetrized, sharpened, masked density of the Mvp1 tetramer, overlaid with the interpreted structure. Shown is a fragment of the BAR α1 helix. **F.** Model-map FSC, as determined by real space refinement protocols implemented in PHENIX. Masked maps were used to calculate the FSC curves for the Mvp1 tetramer (black), the BAR domains (red) and the PX domains (blue).

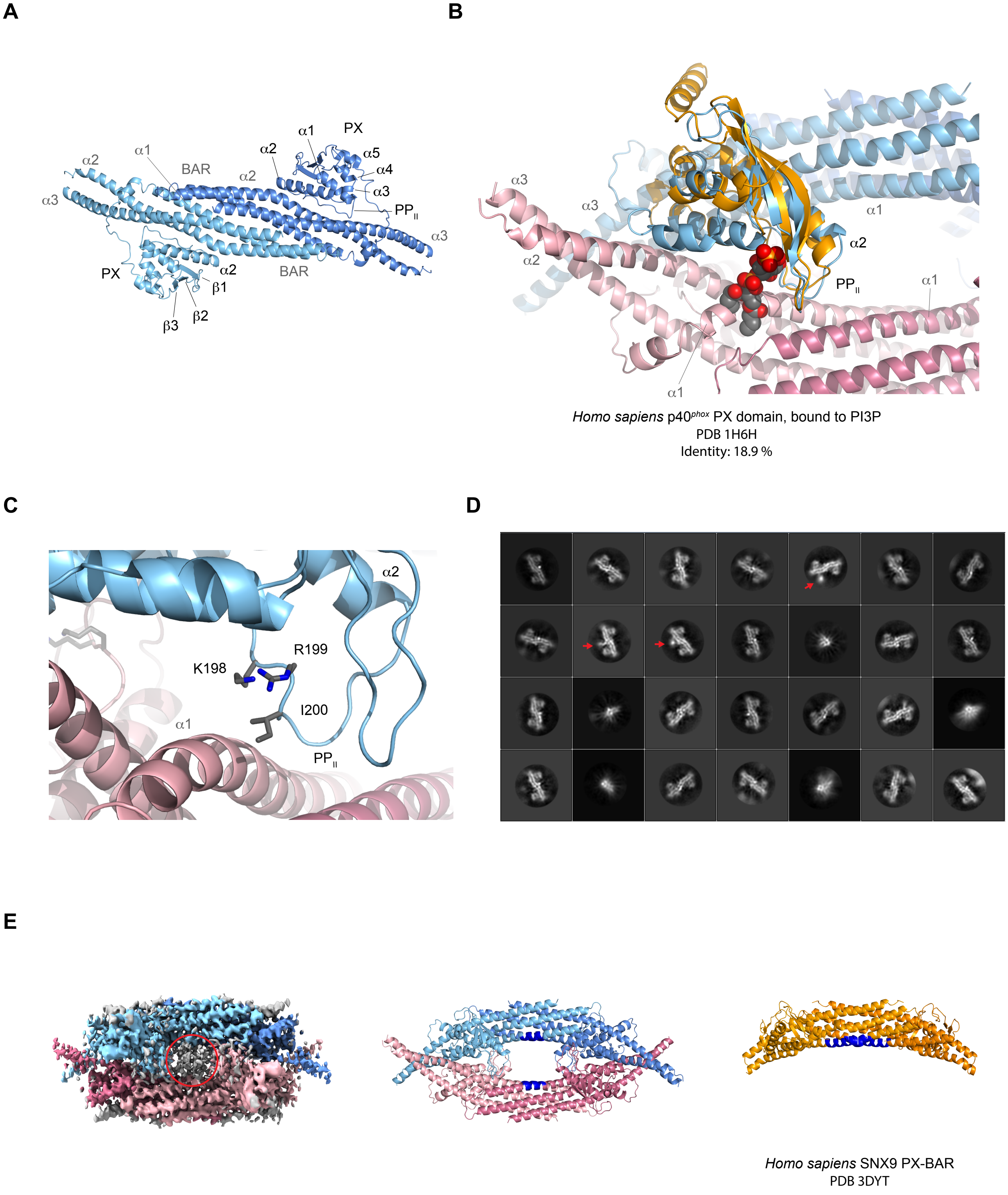

Supplementary Figure 6

Supplementary Fig. 6. Supplementary structure details. **A.** The Mvp1 SNX-BAR dimer, labeled to show the nomenclature of the structural elements in the PX and BAR domains. The view here is toward the convex face of the SNX-BAR dimer. **B.** Superposition of the PI3P-bound PX domain from p40^{phox} onto the Mvp1 SNX-BAR dimer (PDB 1H6H). The backbone superposes with a r.m.s. deviation of Cα atoms of 2.1 Å and the PX domains share a sequence identity of 18.9%. **C.** Location of the residues mutated to generate Mvp1 Mut1. K198, R199 and I200 lie within the Mvp1 PX PP_{II} loop. **D.** Representative 2d class averages of Mvp1 Mut1 particles in different orientations. Note that some particles appear to have an "unleashed" N-terminal domain (red arrows show some examples). **E.** Bridge density contact sites. Left. Bridge density in the D2-symmetrized sharpened map, contoured at 4σ. This is reproduced from Fig. 4E and is colored according to distance from the atomic model of the Mvp1 tetramer: Color is assigned if the density is within 3 Å of the atomic model. The unassigned density is shown in grey. Bridge density is circled in red. Middle. The Mvp1 tetramer, colored as throughout this work. The patches in deep blue depict the regions, on each of the Mvp1 tetramer BAR α1 helices, in closest contact with the bridge density. Right. The crystal structure of the SNX9 PX-BAR module (PDB 3DYT [1]). The patches in deep blue show where the acidic stretch from the N-terminus of SNX9 interacts with the BAR dimer [2].

Supplementary References

1. Wang Q, Kaan HY, Hooda RN, Goh SL, Sondermann H. Structure and plasticity of Endophilin and Sorting Nexin 9. *Structure.* 2008;16(10):1574-1587.
2. Lo WT, Vujicic Zagar A, Gerth F, et al. A Coincidence Detection Mechanism Controls PX-BAR Domain-Mediated Endocytic Membrane Remodeling via an Allosteric Structural Switch. *Dev Cell.* 2017;43(4):522-529 e524.
